# Psychosocial assessment of the family in the clinical setting

**DOI:** 10.1186/s40359-018-0277-5

**Published:** 2019-01-11

**Authors:** Arwa Nasir, Andrea Zimmer, David Taylor, Jonathan Santo

**Affiliations:** 1 Department of Pediatrics, University of Nebraska Medical Center, 982167 Nebraska Medical Center Omaha, Omaha, NE 98198-2167 USA; 2 Boy’s Town Pediatrics, Omaha, NE USA; 3 University of Nebraska Medical Center, Omaha, NE USA; 4 Department of Psychology, University of Nebraska at Omaha, Omaha, NE USA

## Abstract

**Abstract:**

Children develop in the context of the family. Family functioning prominently shapes the psychosocial adaptation and mental health of the child. Several family psychosocial risk factors have been shown to increase the risk of behavioral problems in children. Early identification of families with psychosocial profiles associated with a higher risk of having children with behavioral problems may be valuable for targeting these children for prevention and early intervention services.

**Methods:**

We developed the Family Health Questionnaire (FHQ) for the purpose of evaluating families’ psychosocial risk profiles in the primary care setting. The questionnaire included 10 formative indicators that have been shown to influence children’s behavioral health. We aimed to establish a correlation between the family risk factors on the FHQ and child behavioral health. In addition, we examined the properties of the questionnaire as a screening tool for use in primary care.

Families of 313 of children 4–6 years of age presenting for well child examinations at two primary care clinics completed both the FHQ and the Pediatric Symptom Checklist 17 (PSC-17), a validated screening instrument for pediatric behavioral problems.

**Results:**

We found that the FHQ was positively and significantly correlated with the PSC score (*r* = .50, *p* < .05).

**Conclusions:**

The FHQ may be a valuable screening tool for identifying families with psychosocial risk profiles associated with increased risk of childhood behavioral problems.

## Background

Children develop in the context of the family. Family functioning prominently shapes the psychosocial adaptation and mental health of the child [1]. Adverse childhood events and exposures may result in lifelong negative physical and mental health outcomes [2–4].

Previous literature has documented a number of family variables that are associated with increased risks for adverse child health and behavioral outcomes [5]. These include parental mental health problems [6, 7] parental substance abuse [8], parental conflict [9], domestic violence [10], poverty [11], foster care [12], and parental stress [13, 14]. Identifying families with these psychosocial risk factors can help in targeting services to these families. Interventions aimed at mitigating the negative impact of toxic stress and providing a stable and nurturing environment for infants and young children has been shown to improve health outcomes for children. Parent directed psychosocial interventions [15] and parent training [16, 17] have a significant positive impact on child behavioral outcomes. Strategies for scaling tested and effective family focused preventive interventions are being discussed that aim to promote children’s cognitive, affective and behavioral health [18].

Screening for at risk families in the primary care setting is critical, since for many families, the pediatric primary care office is the only consistent contact with the healthcare system. Screening offers a valuable opportunity to identify families with psychosocial risk profiles associated with an increased risk of psychological morbidity in their children.

Screening tools are increasingly used to identify medical and psychosocial conditions in children. Some of these tools include components to assess family psychosocial factors as part of the total assessment. Examples include the SEEK, a model for prevention of child maltreatment in the primary care setting. The SEEK utilizes a 20 question Parent Screening Evaluation (PSC) tool. The questions on the PSC were validated in relation to the individual factors they measure but not the questionnaire as a whole [19–23]. Another screening instrument, the Survey of Wellbeing of Young Children (SWYC) is a 54 item milestone-based developmental questionnaire that includes questions to assess some family factors. The family assessment portion in the SWYC is brief, and has not been individually validated [24].

Given the evidence of the importance of the family environment on childhood health outcomes, the AAP Task Force on the Family recommends that pediatricians expand their practices to encompass the assessment of family structure and function [25]. However, in clinical practice, there are a number of important barriers to the implementation of this recommendation. These include health care delivery models and reimbursement structures that do not reward attention to psychosocial and behavioral issues. This reduces the time available to the clinician for assessment of these problems [26]. Another barrier is the lack of training among pediatricians in psychosocial issues and screening [27].

The questionnaire was developed by the authors as a screening tool to identify families having psychosocial risk profiles associated with childhood behavioral problems in the primary care setting. It explores 10 formative indicators that have been identified as being causally linked to adverse childhood behavioral outcomes. These indicators based on extensive review of the literature on this topic. These indicators include a history of childhood adversity in the parent, poor social support, a fragile family structure, parental mental health problems and substance abuse, geographic instability, domestic violence, poverty, as well as parental conflict and stress. The relevant literature indicates that these factors capture the factors most closely associated with adverse childhood experiences, and also provide a global evaluation of the family’s psychosocial milieu [5, 10–12, 14, 20, 28–31].

The instrument was translated to Spanish by a bilingual and bicultural (American/Hispanic) member of the institution’s interpreter services office. To address issues of content and semantic equivalence as well as the cultural and conceptual aspects of the instrument translation, the questionnaire was independently reviewed by an additional 3 trained bilingual interpreters. Several other adjustments were made to the questionnaires based on their input.

Subsequently, the instrument was pilot tested with 3 bilingual health care workers (nurses and receptionists) and 3 bilingual parents. Based on their input, no further modifications were needed.

In a previous study, the 10 item FHQ was pilot tested on 55 families. A significant correlation was found between the FHQ and Pediatric Symptom Checklist 17, (PSC-17), a validated pediatric behavioral screening tool [32].

The aim of this study was to confirm the correlation between the FHQ as a measure of family psychosocial risk and PSC-17 as a measure of the behavioral wellbeing of the child [33]. We also aimed to examine the properties of the FHQ to determine its validity as a screening test.

A secondary aim was to explore the correlation between the FHQ and parental perceptions of the health status of their child.

## Methods

### Study participants

Parents of 315 children between 4 and 6 years of age presenting for kindergarten physical examinations or other health care maintenance visits were recruited from two primary health clinics in Nebraska from June 10 to August 10, 2016.

### Sample size

We estimated a sample size of 300 subjects based on literature indicating that this number of subjects is generally acceptable for internal validation of psychiatric scales [34]. We obtained permission from the IRB to recruit 315 patients to allow for potential withdrawals or exclusions.

### Inclusion and exclusion criteria

Parents of all children who had appointments for kindergarten physical examinations during the study period were recruited. Exclusion criteria included foster families, because some foster parents had limited knowledge of the family history or the child’s behavior because of recent placement. Children accompanied by a non-guardian were also excluded. Parents who spoke other languages than English or Spanish were excluded. Translation to other languages other than Spanish was deemed to be impractical for the purposes of this study due to the low numbers of these patients.

### Ethical considerations

The University of Nebraska Medical Center Institutional Review Board approved the protocol.

### Study procedures

Informed consent was obtained from the parents, and they were asked to fill out the FHQ and PSC questionnaires as well as answer questions pertaining to their perceptions of their child’s health in the past year. Data obtained from the electronic medical record included the child’s current BMI and the number of sick visits to the ED or the primary care office in the past year. The study was not powered to detect a difference in the number of ED or office visits, therefore, the analysis of this variable is not included in the results. The surveys were available in English and Spanish.

The Pediatric Symptom Checklist is a brief version of the Pediatric Symptom Checklist 35 (PSC-35). PSC-17 is a parent self-administered questionnaire that explores a range of behavioral symptoms in children. It includes 3 subscales for internalizing, externalizing and attention deficit symptoms. A score of 15 or more suggests the presence of significant behavioral or emotional problems. In a large study using data collected on 80,680 pediatric outpatients, ages 4–15 years, over 10-year study period, PSC-17 showed high reliability and was comparable to the original instrument. The study supported the use of the PSC-17 in clinical practice and research [35].

The Family Health Questionnaire is shown in Table 1. Questions have dichotomous answers of yes or no. The answers were scored as 0 or 1, where 0 indicated the presence of a risk factor. A score of 10 indicates the absence of risk factors and the lower the score the greater the number of risk factors present.

**Table 1:** Please answer the following questions **about yourself**. Por favor conteste las siguientes preguntas **sobre usted mismo**

	Yes Sí	No
Did you have a happy childhood?		
¿Tuvo una niñez feliz?		
Have you been living in the same place for more than 2 years?		
¿Ha estado viviendo en el mismo lugar por más de 2 años?		
Do you have friends and family who care about you?		
¿Tiene amistades o familiares que se preocupan por usted?		
Does your child live with both biological parents?		
¿Su hijo vive con los dos padres biológicos?		
Do you have a good relationship with your partner or spouse?		
¿Usted tiene buena relación con su pareja o conyugue?		
Have you ever been diagnosed with a mental health problem?		
¿Usted ha sido diagnosticado con un problema de salud mental?		
Do you have a history of substance use (drugs, alcohol)?		
¿Usted tiene antecedentes de uso de sustancias (droga, alcohol)?		
Do you have financial difficulties (money problems)?		
¿Usted tiene dificultades económicas? (problemas con dinero)?		
Have you experienced domestic violence?		
¿Usted ha sido víctima de violencia domestica?		
Do you always feel stressed?		
¿Siempre se siente estresado?		

Would you like to speak to our patient care coordinator to help you get a referral for psychological or social services?

## Results

From June 22–October 15^,^ 2015, we recruited 315 families from 2 primary care pediatric clinics. One family declined to participate citing time constraints, and one of the questionnaires was excluded because of concerns regarding question comprehension due to language barrier. A total of 313 data points were available for analysis. All the parents filling out the questionnaires were mothers or parents together.

The study sites were an urban academic, hospital-based general pediatric clinic and a community clinic which is outside the metropolitan area. Around 80% of patients attending each of the clinics are publically insured. Table 2 describes the demographic features of the participants.

**Table 2:** Patient Characteristics

	Frequency:	Proportion
N	313	
Sex (Male)	161	51.4%
Primary Insurance:		
Public Insurance	201	64.2%
Private Insurance		28.1%
No Insurance	24	7.7%
Spanish Surveys	23	7.3%

In our sample, 47.3% of children were not living with both biological parents. This compares to a nationwide rate of approximately 35% in 2015 [36]. Nearly a fifth of mothers (18.8%) in our sample reported having experienced domestic violence, a rate consistent with national estimates [37]. Most mothers in our sample (92.1%) reported a good relationship with their current spouse or significant other. A history of mental health problems was reported by 12.8% of mothers in the sample. This compares with national data indicating that 4.2% of US adults suffer from serious mental illness and 18.1% having any mental illness [38].

The median FHQ score was 8, indicating the presence of 2 risk factors, with a range from 2 to 10. The FHQ score was ≤7 in 26% of families indicating 3 or more risk factors ≤6 in 12.6% of families, indicating 4 or more risk factors.

The median PSC-17 score was 8, with a range of 0–24. Twenty five children (8%) scored 15 or above on the PSC which is the cutoff score for a positive PSC screen.

We also tested for differences in PSC-17 scores based on positive responses to the individual FHQ items. Table 3 shows the frequencies of responses to the questions. 24.3% of parents reported that they have financial difficulties, and 16.3% reported that they feel always stressed.

**Table 3:** Frequency of responses to FHQ and group differences in PSC-17 scores

	Freq. / %	Mean Difference in PSC	t (df)
FHQ 1 - Happy childhood	283 (90.4%)	1.44	1.59 (311)
FHQ 2 – Same home for 2 years	213 (68.1%)	2.34	3.86 (160.36)*
FHQ 3 - Friends and family support	312 (100%)	NA	NA
FHQ 4 - Live with both parents	165 (52.7%)	1.77	3.31 (266.07)*
FHQ 5 – Good Parental relationship	280 (92.1%)	2.58	1.76 (24.60)
FHQ 6 - History of mental health problems	40 (12.8%)	−2.86	3.65 (311)*
FHQ 7 - History of substance abuse	13 (4.2%)	NA	NA
FHQ 8 - Financial difficulties	76 (24.3%)	−2.12	3.45 (310)
FHQ 9 - Domestic violence	59 (18.8%)	−2.59	3.88 (311)*
FHQ 10 – Stress	51 (16.3%)	−4.40	6.46 (311)*

*p < .05

An exploratory factor analysis was conducted to create a latent factor model of the FHQ (with categorical indicators) using M-Plus (ver. 7.2, Muthen & Muthen, 2016). Two questions on the FHQ showed no variability and were excluded from the factor analysis. These were: “I have family and friends that care about me” which was answered unanimously in the affirmative. The other question was pertaining to a history of substance abuse. Only 13 individuals (4.2% of the sample) answered affirmatively. It is possible that disclosure of a history of substance abuse may have been problematic for many parents. The findings revealed that a single factor solution was a good fit to the data (Δχ^2^_(238)_ = 201.73, *p* = .96) and significantly better fit to the data than the two factor solution (Δχ^2^_(7)_ = 37.25, *p* < .001). Neither the three factor (Δχ^2^_(6)_ = 10.26, *p* = .11) or four factor (Δχ^2^_(5)_ = 8.57, *p* = .13) solutions resulted in a significantly improved fit. The factor loadings for the single factor model were all significant and positive providing an estimated reliability of .793.

**Fig. 1:**
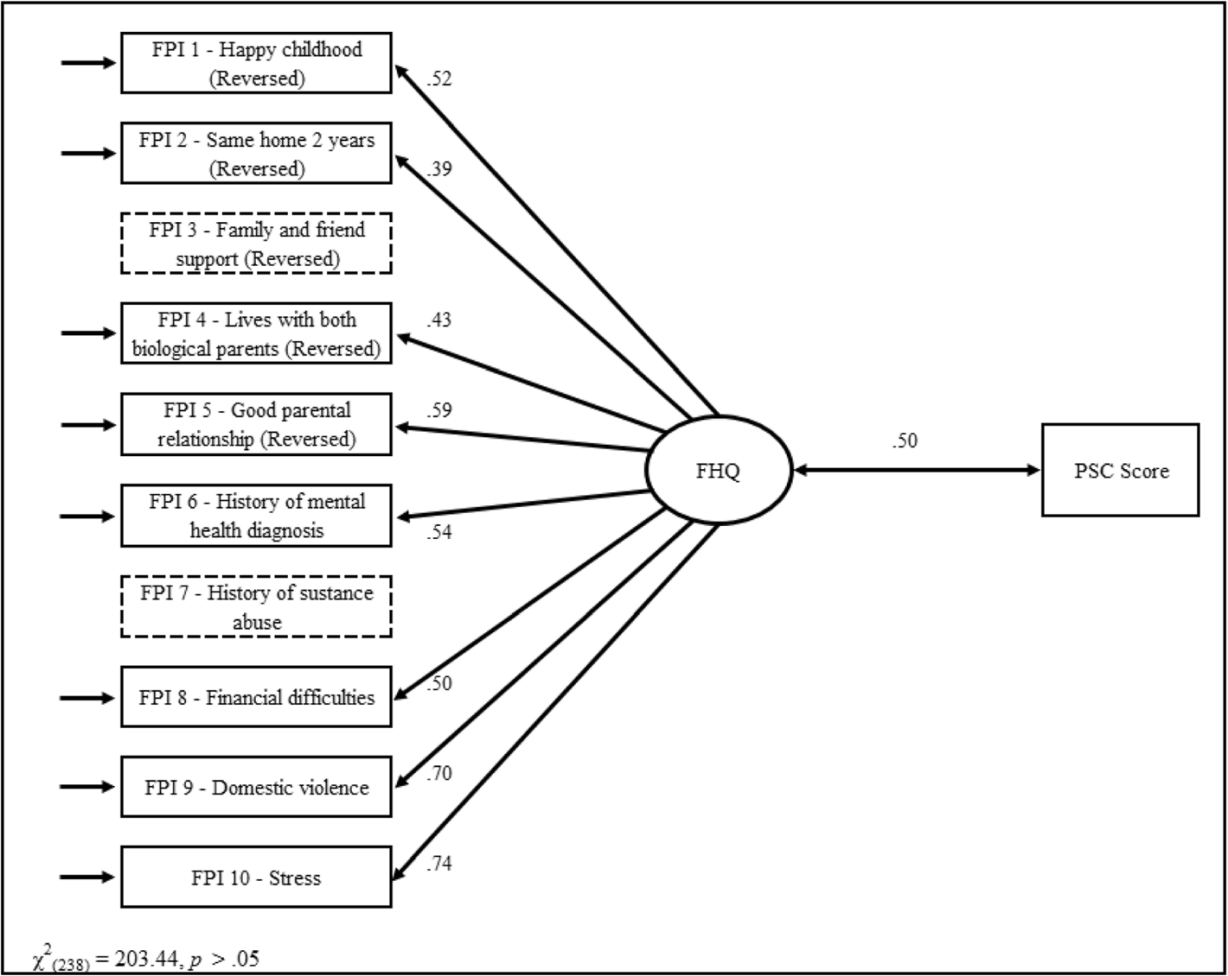
Factor analysis

**Fig. 2:**
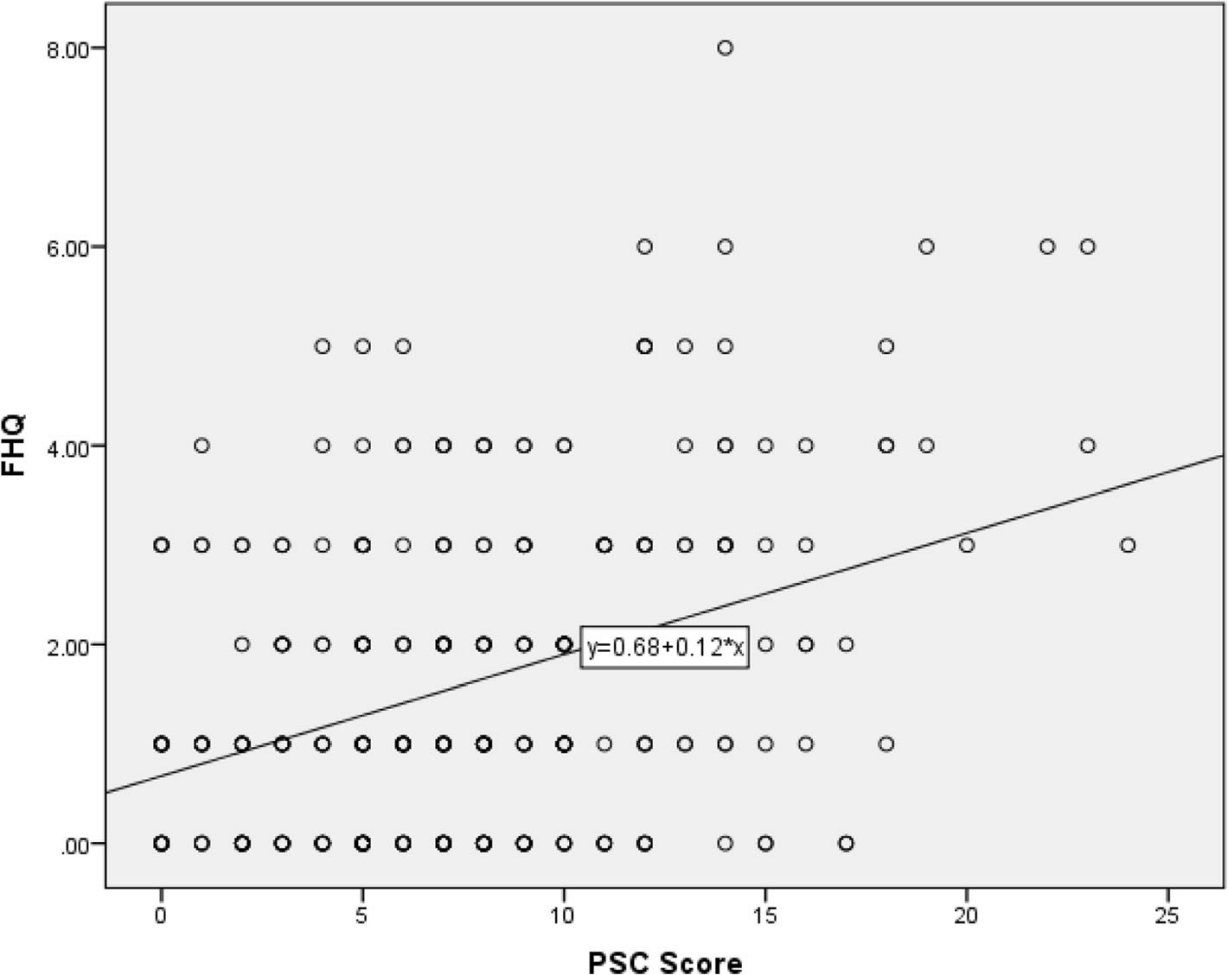
scatter plot of correlation between FHQ and PSC scores

Following the exploratory model, a single factor confirmatory factor analysis was created [Fig. 1]. There was a significant positive correlation between the total score for the remaining 8-question FHQ and the PSC scores, (*r* = .50, *p* < .05; see Fig. 2). The resulting model was a good fit to the data (χ^2^_(238)_ = 203.44, *p* = .95).

Table 3 also describes the individual differences in the response to the FHQ with PSC scores. The largest difference (of 4.40) was seen with FHQ10 (“I always feel stressed”; t(311) = 6.46, *p* < .001). Similar significant differences were observed for FHQ2 (“Same home for 2 years”; t(160.36) = 3.86, *p* < .001), FHQ4 (“Live with both parents”; t(266.07) = 3.31, *p* = .001), FHQ6 (“History of mental health problems”, t(311) = 3.65, *p* < .001) and FHQ9 (“Domestic violence”; t(311) = 3.88, *p* < .001).

Lower FHQ scores (indicating a higher number of risk factors) also correlated with parents’ perception of their child’s health as poor (*r* = −.12, *p* = .04). FHQ scores also significantly differed as a function of insurance status (F_(2, 310)_ = 16.85, *p* < .001, η^2^ = .10). Families with private insurance had significantly higher scores (lower numbers of risk factors, M = 9.11, S.D. = 1.20) than families with public insurance (M = 8.03, S.D. = 1.56) or non-insured families (M = 8.38, S.D. = 1.35). Lower FHQ scores were also associated with higher BMI, but the association did not achieve statistical significance (r(311) = −.09, *p* = .11).

## Discussion

Screening for family psychosocial risk can identify families who may benefit from interventions directed at improving childhood health outcomes. In this study we documented a strong correlation between a newly developed family psychosocial health questionnaire, the FHQ and behavioral problems in children measured by the PSC, a validated childhood behavioral health symptom instrument.

Lower FHQ scores were also correlated with parent’s perception of poor health in their child. This is a subjective measure of the effect of psychosocial risk factors on child’s health. Although the study was not powered to detect significant BMI correlations, we collected BMI measurements from the medical records. The correlation between lower FHQ and higher BMI did not reach statistical significance. Further studies to explore the correlation between FHQ and child health outcomes and healthcare utilization would be needed.

Significant positive correlation was found between lower FHQ scores (more risk factors) and public insurance status of the family. Public insurance is a marker of low income and economic disadvantage which has been correlated with adverse childhood health outcomes [39–42].

We also documented the feasibility and acceptability of administration of the FHQ in a sample in the primary care setting. The test was self-administered by the caregiver, required no training, and took less than 2 min to complete on average. We believe this FHQ can be very useful in screening for family psychosocial risk in primary care.

This study also documented the prevalence and profile of psychosocial risk in the population sample, identifying strong correlations between certain psychosocial risk factors such as poverty and parental mental illness and child behavioral health.

Family psychosocial factors contribute to the toxic stress that is an important risk factor for childhood psychopathology. Interventions in early childhood programs that aim to reduce toxic stress have been shown to improve health outcomes, improve learning, decrease achievement gaps, and boost future earnings [43]. The early identification of families of children at risk for behavioral problems may offer an important opportunity to mitigate negative behavioral outcomes.

The primary care setting is ideal for screening for family psychosocial risk because of the frequent longitudinal encounters with families of small children.

### Study limitations and future directions

Both the FHQ and PSC-17 are self-reports by the same parent, which raises the issue of common reporter bias. However, in this situation, the reporter’s perception, even if biased, is important. Future validation with larger and multicenter samples using other objective evaluations of child behavioral health may be helpful. Of course, correlation of the FHQ with the presence childhood behavioral problems does not prove causation. However, others have established causal effects of early childhood adverse events on negative health outcomes, many of which are included in the FHQ.

This tool was tested in a population with significant burdens of adversity such as poverty and other psychosocial risks. Further testing of this tool in other populations with other socioeconomic and demographic characteristics would be important to determine generalizability of this tool to other populations. Future work should also explore the use of this instrument in the scaling of family focused interventions aimed at preventing behavioral problems.

## Conclusions

Children live in the context of the family. Any effort to address the psychosocial environment of the child must address family resources and psychosocial risk factors.

The FHQ is a quick and easy to use screening tool that may be helpful in identifying families with increased psychosocial risk for child adverse outcomes. Identifying families who are at higher risk for family dysfunction leading to increased psychosocial risk among children could help target resources for further evaluation and intervention. Early identification, paired with prompt and effective intervention might help to reduce childhood exposure to adverse environments, reducing the physical and mental health impacts of these environments, improving wellbeing and optimizing potential [18].

## Abbreviations

AAP: American Academy of Pediatrics
FHQ: Family Health Questionnaire
PSC-17: Pediatric Symptom Checklist-17
